# The Dictionary of Foods and Drinks

**Published:** 1855-12

**Authors:** 


### THE DICTIONARY OF FOODS AND DRINKS;

### A COMPENDIUM OF ALIMENTARY SUBSTANCES IN USE THROUGHOUT THE WORLD: THEIR HISTORIES, DIETETIC PROPERTIES, AND ADULTERATIONS.

### PART III.

ADYNAMON WINE, wine without strength. A drink taken by the Roman ladies. It was made by boiling ten pints of water in twenty pints of white wort.

AERATED WATERS. Waters highly charged with a gaseous body are said to be *aërated.* They are largely produced by nature, and also artificially prepared. Strictly speaking, all waters are more or less impregnated with some form of gas. The gaseous substances most frequent are carbonic acid, oxygen, nitrogen, and sulphuretted hydrogen. Of these, carbonic acid, owing to its great solubility, is present most abundantly. Oxygen, being less soluble, is found in smaller quantities, and nitrogen in proportions smaller still. However, some springs hold in solution liberal proportions of these gases. Certain special springs also contain sulphuretted hydrogen, and are hence denominated aërated springs. Under the ordinary pressure of the atmosphere, 100 cubic inches of water will dissolve from two to four cubic inches of atmospheric air, its own bulk of carbonic acid, and a like measure of sulphuretted hydrogen; but saturated solutions of the two last are not often met with. The springs of Knaresborough and Matlock possess a notable quantity of free carbonic acid, while the Buxton water is remarkable for the sulphuretted hydrogen it holds in solution: the Saratoga Congress spring in America contains 114 cubic inches of *fixed air* to the 100 of water; and that of Pyrmont in Germany affords no less than 160 of the same gas.

In springs arising from volcanic soil, *sulphurous acid* is sometimes to be found.

Artificial aërated waters may be considered under two heads; 1st, in which the gas is generated originally in the water itself, either by a sort of fermentation, or by the simultaneous addition to it, in a firmly closed vessel, of an acid and a carbonated alkali; or, 2ndly, by evolving carbonic acid in a separate chamber, by any suitable means, and afterwards forcing it with a double or treble pressure into water, containing, or not, as the case may be, a little carbonate of soda or potassa. When properly secured in strong bottles, these waters may be kept undeteriorated for a long period.

The chief representatives of these two methods of manufacture are, respectively, those popular beverages ginger beer and soda water. When aërated water is released from pressure, the excess of gas confined in it is quickly liberated in numerous small bubbles, producing the refreshing phenomenon we call *effervescence.*

Occasionally hydrosulphuric acid gas and various chemical salt- are added to water for medicinal purposes with the view of imitating the natural springs. Aërated water can hardly be said to be adulterated, as the few impurities found exist in the materials employed: vinegar and oil of vitriol, it has been asserted, replace the tartaric and citric acids in some samples, but this fraud is very doubtful. In a moderate quantity effervescing waters are cooling or beneficial; and nascent carbonic acid is said to assist the digestion.

AGARIC, a genus of *Fungi*, containing numerous species, growing on trees, or springing from the earth: of the latter, some are used as articles of food; others are poisonous. The common edible mushroom is the *agaricus campestris*. See *Mushroom.*

AGAVE AMERICANA, the American, or Mexican *Aloe*, natural order *Liliaceæ;* called also the *Maquey.* The fermented sap is used in Mexico to make large quantities of agave wine, which is sometimes called pulque and octli. The Agave Americana grows to a height of eight or ten feet. The sap or juice yielding the wine is extracted from the stem.

AGAVE WINE, a Mexican wine, obtained from the *Agave Americana.* The process of manufacture is tersely described by Professor Johnston. In the plantation the Indian watches each plant as the time of its flowering approaches; and just when the central shoot or flower-stem is about to appear, he makes a deep cut and scoops out the whole heart *(el corazon)*, or middle parts of the stem, leaving nothing but the outside rind. This forms a natural basin or well, about two feet in depth and one and a half in width. Into this well the sap, which was intended to feed the shoot, flows so rapidly that it is necessary to remove it twice, and sometimes three times a day. The sap has a sweet taste, ferments spontaneously, and the old fermented juice soon induces fermentation in that which is newly drawn. After twenty-four hours fermentation it becomes wine fit for drink. A good maquey yields from eight to ten pints a day, and often for two or three months. In the course of the fermentation three products are formed—viz., alcohol, an acid, and an ingredient which gives off a most disgusting smell, resembling the smell of tainted meat. The nature of the acid or of the disagreeably smelling substance is not known. The agave wine resembles cider in taste; and when the disgusting smell is got over, it is much relished as a drink. It is intoxicating; but many medicinal virtues are ascribed to it.

AGOUTI, an animal peculiar to South America and the West Indies. It belongs to the order *Rodentia.* The Agouti resembles the rabbit in many particulars. It is of about the same size, and burrows its home. In eating, however, it holds its food like a squirrel—in its paws. It lives on vegetables. It is much esteemed as an article of food in the Antilles and other parts where it is native. The flesh is white, and resembles that of the rabbit.

AGUARDIENTE. (Spanish, *agua ardiente*, hot water). A term used for brandy and for some other alcoholic drinks of the liqueur class. It is much used in Spain, and in many tropical countries. In composition, aguardiente differs much in different localities. Sometimes the term is applied merely to brandy; sometimes, and especially in Spain, to a mixture of brandy, honey, and aniseed, exposed for some time to the action of the sun and air. This compound resembles what is called aniseed brandy in this country. In Mexico, the term aguardiente is applied to the ardent spirit obtained by the distillation of the fermented juice of the Agave Americana.

The action of aguardiente on the body is mainly due to the alcohol it contains, whatever may be the process of manufacture. Its frequent use in Africa is the cause of many diseases of the digestive organs, according to Dr. W. F. Daniell.

AGUAMIEL. (Spanish, *agua*, water; *miel*, honey; honey water). A name applied to the sweet sap which exudes from the *Agave Americana* before fermentation is set up. Aguamiel, as the name implies, has a sweet taste, and has none of the disagreeable odour so peculiar to it after fermentation. It soon ferments spontaneously. *(Johnston.)* Aguamiel itself is not generally used as a beverage.

AI WINE, a Champagne wine, very famous in the time of Henry VIII. It was made at Ai, a canton of Champagne; hence the name.

AKEE, the fruit of *Cupania Akeesia*, a plant of the natural order *Sapindaceæ*. It is a native of Guiana, and was carried thence by Captain Bligh, in 1793, to Jamaica, where it is used as an article of food: the slightly acid arilla, at the end of the seed, being the part eaten.

ALBAN WINE *(Vinum Albanum)*, a wine used by the ancient Romans, and referred to by Pliny.

ALBATROSS, Eggs of. The Albatross is a large aquatic bird, belonging to the order *Palmipedes* of Cuvier. The giant Albatross is common in the Austral seas, where it feeds on flying fish and mollusks. It builds a nest of earth, and lays large numbers of eggs, which are excellent food.

ALBUMEN, or Albumine (*albus*, white); a very important azotised nutritive principle, widely diffused throughout the animal and vegetable kingdoms.

The white of egg presents a specimen of albumine nearly pure, and the serum of blood also contains a large quantity of this substance in solution. From either of these sources it may be precipitated in light flocks by exactly neutralising the liquid with acetic acid, and largely diluting with cold distilled water: thrown upon a filter, washed and dried at a temperature not exceeding 120° Fahr., this precipitate offers albumine in a convenient form for studying its reactions and properties. Thus prepared, pure albumine is a yellowish-white, tasteless, amorphous powder, insoluble in water and alcohol, but readily dissolving in the former, if a trace of alkali is also present: from this solution it can be thrown down by heat, from 140° to 160° being generally sufficient for the purpose; but the coagulated albumine is easily redissolved in a solution of potassa or soda, and is distinguished from the same substance dried at a lower temperature by not being soluble in a strong solution of nitrate of potassa, which dissolves ordinary albumine with great ease. The mineral acids and most of the metallic salts also precipitate albumine; the efficacy of white of egg, as an antidote for corrosive sublimate, depends upon this property, the insoluble compound formed being a combination of *chloride of albumine* and *chloride of mercury.* Alcohol added in excess, tannic acid, or infusion of galls, likewise precipitate albumine.

According to Mulder, albumine is composed of

| | |
|---|---|
| Carbon | 53·5 |
| Hydrogen | 7·0 |
| Nitrogen | 15·5 |
| Oxygen | 22 0 |
| Phosphorus | 0·4 |
| Sulphur | 1·6 |
| | 100·0 |

The presence of free sulphur is well shown by the blackening of a silver spoon when left in contact with a boiled egg—a circumstance familiar to every one, and caused by the formation of the sulphide of the metal;—and if albumine in any form is boiled in a dilute solution of potassa, and the liquid is filtered, neutralized, and mixed with a little acetate of lead, the characteristic black precipitate of sulphide of lead is immediately thrown down. Phosphorus cannot be detected in albumine quite so easily; but if white of egg be evaporated to dryness several times with concentrated nitric acid, until the organic matters are wholly volatilised, and the residue be dissolved in hydrochloric acid, the addition of a mixed solution of sulphate of magnesia, chloride of ammonium, and free ammonia, will cause the ammonio-phosphate of magnesia to fall in a crystalline form.

The important agents of nutrition, *Fibrine* and *Caseine,* are remarkable for the close analogy they bear to albumine, in properties, in composition, and in general character: indeed, so little do their analyses differ, that the three may be considered isomeric. Fibrine exists in solution in the blood of all animals during life, coagulating after death to form, with blood-corpuscles, the *crassamentum,* or clot. Caseine is an ingredient of milk, the part called the "curd" consisting almost entirely of this principle. These two substances comport themselves like albumine with chemical reagents. Caseine, however, is precipitated by acetic acid, and is not coagulable by heat, these being about the only exceptions to the general rule.

Albumine, fibrine, and caseine, all have their doubles, so to speak, in the vegetable world; for if dough made from wheaten flour be carefully washed upon a fine sieve, a milky liquid will pass through, which, on becoming clear in a little time by depositing a quantity of starch, leaves vegetable albumine in solution. Vegetable albumine is also contained in many vegetable juices, as in that of the potato; and, as the writer has found, in apples. If the sieve be examined after the above experiment, there will be found remaining in it a greyish adhesive body called *glutine;* this, digested in alcohol, and then in ether, which dissolve out some *gliadine* and a little oily matter, will be found perfectly indistinguishable from the fibrine of blood. Peas, beans, almonds, and other seeds of a like nature, yield a considerable amount of vegetable caseine, possessing all the properties of that obtained from milk. See also *Fibrine, Caseine, Cheese,* and *Milk,* in the *Dict. of Foods and Drinks.*

When either albumine, fibrine, or caseine, from any source, is dissolved in a moderately strong solution of potassa, and the liquid is maintained at about 130° for some time, then filtered, and acetic acid added in excess, a little sulphuretted hydrogen is evolved, and a white gelatinous precipitate falls of the much discussed substance, proteine: a substance, the discoverer of which—Mulder—first asserted to be free from sulphur and phosphorus; albumine, fibrine and caseine being most probably direct combinations of proteine with the above elements. The detection of sulphur, however, in proteine, soon rendered this theory untenable; and Mulder now suggests that the proteine compounds may be combinations of that substance with *sulphamide* and *phosphamide.* There are chemical objections to this theory.

Albumine, in common with most other direct products of the animal and vegetable economy, may be preserved indefinitely in the anhydrous state, but is speedily decomposed under the joint action of air and moisture, a remark which applies to the other proteine compounds. It is a simple and highly nutritious article of food, of easy digestion, and supplying azotised or structure-making substance. It is best as food when coagulated by heat, as in the boiled egg.

Closely allied to albumine, are several other of the so-called proteine compounds, such as vitelline, globuline, hæmatosine, legumine, glutine, and diastase.

VITELLINE constitutes the chief solid material of the yolk of eggs, being present to the extent of 15 to 17 per cent. It differs from soluble albumine in composition, by containing three equivalents more of the elements of water, and is analytically distinguished from it by its non-precipitation on the addition of solution of lead or copper.

In the soluble form, vitelline has not been obtained pure; but in the coagulated state, it can be had quite free from all extraneous matter.

GLOBULINE. The *crystalline lens* in all animals consists of a nearly pure solution of this substance, which very greatly resembles albumine, and which, together with hæmatosine in unknown proportions, forms the fluid contained in the blood-corpuscles.

Globuline will not coagulate at a lower temperature than 164° F., and may therefore be easily separated from albumine.

HÆMATOSINE is believed to be the colouring principle of the blood, and is by some classed among the proteine compounds, although essentially differing in composition, the hydrogen being deficient and the carbon greatly in excess; still, in general properties, hæmatosine offers many points of resemblance to albumine; but as it has never been obtained in a pure state, its precise composition, and its function—probably an important one—in the animal economy, are not known.

GLUTINE, or Gluten, is in reality a mixture of *gliadine* and vegetable fibrine.

Of DIASTASE very little is known. It will be treated of under the head *Malt.*

The following substances are considered to be in some measure derived from the proteine compounds:

GELATINE, and its second modification, *Chondrine;* FIBREINE, the basis of silk, sponge, and the delicate thread fabricated by the common spider; and CHITINE, a whitish product, obtained by boiling the elytra of the cockchafer or other similar insect, successively with water, alcohol, ether, acetic acid and caustic potassa.

ALCOHOL, (the Arabic article, *al;* and *kohol,* a grinding or trituration, by which any substance is finely divided), called in common parlance, spirit of wine; a volatile spirituous fluid, of the specific gravity of 0·7938 at 60° Fahr. When anhydrous, *i.e.*, free from water, alcohol is colourless, and emits a spirituous odour. It boils at 173° Fahr. The elementary composition of alcohol is four atoms of carbon, six of hydrogen, and two of oxygen. It is highly inflammable, and burns with a bluish flame, free from smoke. It has never been frozen. Commercially there are several varieties of alcohol, viz., "unsweetened gin", proof spirit, etc., which are merely alcohol diluted more or less with water. Proof spirit must contain 49¼ per cent. of alcohol. Alcohol is derived from grape-sugar by a natural process of fermentation. The sugar, in its original form, is composed of 24 atoms of carbon, 28 of hydrogen, and 28 of oxygen. When this sugar, in solution with water, is brought into contact with putrescent asotized materials, as putrid blood, white of egg, or common yeast, the sugar is resolved into alcohol with the evolution of carbonic acid. By repeated distillation, the alcohol is driven over free from the water.

Alcohol imparts specific, exhilarating, and intoxicating properties to ales, wines, and spirits. The proportion of absolute alcohol in any given specimen of spirit may be obtained by simply taking the specific gravity of the spirit. A spirit which contains one per cent. of alcohol will give at 60° Fahrenheit, a specific gravity of 0·9981, while one containing 30 per cent. of alcohol will give a specific gravity of 0·9578. As constituting the most common drink except water, alcohol holds an important place as an article of life. It belongs to the combustible class of foods, those, that is to say, which are burnt for the purpose of supplying animal heat. Alcohol, therefore, is a food, and in moderate quantities is useful as such. In excessive proportions it acts first as a stimulant, and then as a depressant on the system. Its peculiar action in very large doses is to arrest the oxidation of the animal tissues, and produce insensibility. However received into the body, whether it be breathed into the lungs in the form of vapour, or taken into the stomach as a liquid, it is absorbed, though much more quickly when received by the lungs, and gives rise to its special symptoms. Drunkenness arising from alcoholic drink is a state strictly analogous to the narcotism arising from chloroform: and during the drunken state the chemistry of life is seriously retarded. The body takes in less oxygen and gives out less carbonic acid. The substance, however, being volatile, is in time evolved from the system, either directly or indirectly, upon which recovery gradually takes place. The long continued free use of alcohol as a beverage leads to great changes in the blood, and in the organs of the body. The pathological changes induced are, 1. cirrhosis, a peculiar condition of the liver, in which that organ becomes firmer and harder in structure than is natural, and of a roughened nodulated appearance, called gin-drinkers' liver. 2. Degeneration or softening of the heart and other muscles, often from fatty transformation. 3. Modifications in the organisation of the brain substance, giving rise to delirium and often to permanent insanity. The form of insanity peculiar to the effects of alcohol has been called by psychologists oinomania, and is supposed by some to be hereditary. 4. Alcohol has the property of rendering the blood fluid, uncoagulable, and wanting in plastic power. 5. The kidneys may pass through a fatty transformation, giving rise to the elimination of albumen in the urine, and to a class of symptoms which are in the end incurable. Thus alcohol in immoderate proportions is a very dangerous agent. In moderation there is, however, no valid physiological objection to its use.

The injurious effects of alcohol on the body vary somewhat according to the spirit in which it is taken. Whiskey leads more commonly to kidney disease; gin to liver disorder; beer to fatty degeneration of the muscles; rum to dyspepsia. Water is the chief adulterant of alcohol: the specific gravity is the best test for this fraud.

ALDER (Saxon *alor*), the common name for the *Betula Alnus*, a tree of the natural order *Betulaceæ*, Linnæan class and order *Monœcia Polyandria*. When bored in the spring, the tree yields a saccharine juice, which on being fermented, yields a liquid termed Alder wine.

ALE (Saxon *ealu*), a beverage known from the most ancient times. It was made by the ancient Egyptians, was possibly drank by the Greeks, and was in the early period of the history of Gaul a common beverage. Amongst the Saxons and Normans it was much esteemed, and its present name is of Saxon origin, "ealu."

Ale is an infusion of malt, which is put through the process of fermentation by yeast, and afterwards flavoured with hops. Ales are of different kinds, but essentially there is no difference except in matter of quality, colour, or flavour. The difference in colour has given rise to the names of brown ale, pale ale, etc. The variety of colour should strictly depend on the character of the malt used; high dried malt giving the brown, and lightly dried giving the pale variety: but sometimes colouring matters are added to produce the brown effect.

The distinction betwixt *ale* and *beer* depends mainly on the amount of hops used, ale containing the larger flavour of hops. In some districts, however, the term "beer" is simply used to express a weaker variety of ale, a second infusion of the original mash. Again, the term beer is occasionally used in a generic sense to express malt liquors of all kinds. Bitter ale is a variety of ale in which the bitter principles exists in great quantities, and in the manufacture of which pale malt is used. Into the composition of ale there enter starchy matter, lupuline (the aromatic bitter principle of the hop), extractives, lactic acid, various salts, and free carbonic acid and water. The freshness of ale depends on the presence of carbonic acid, its tartness on the presence of acetic or lactic acids. Various names are applied to ales, simply to distinguish the locality where they are manufactured, as Burton ale, Edinburgh ale, etc. The celebrity of any special ale rests rather on the taste of the ale drinker than on any special distinction in the nature of the ale itself. The specific gravity of ale varies from 1·02 to 1·0055.

As adulterations of ale, Chevallier gives the following:—acetate of copper, lead, tartaric acid, alum, as accidental products; burnt chicory, lichens, leaves and bark of box, flowers of buckbean, lime-flowers, gentian, poppy-heads, guiacum wood, juice of balm, as substances added to give colour; and henbane, grains of paradise, cocculus indicus, cloves, pellitory, and ginger. Strychnine has also been thought to be an adulterant of ales, being added to give a bitterness in place of hops; but this does not seem to have been fully proved. The same remark applies to colocynth, which is also said to be sometimes employed for the same purpose, and to an acid called picric (which is derived from indigo), aloes, and benzoin. Sulphate of iron is a more certain and common adulterant. Of these adulterants colocynth and the leaves and bark of box possess purgative properties, and are therefore most injurious.

Picric acid is probably astringent. For the action of the other adulterants see *Adulterants.*

In moderate quantities, pure ale is a nutritious and wholesome beverage. Its stimulating properties are due to the presence of alcohol. As a food it supplies the elements which go to support the animal temperature—the respiratory or combustible elements. Taken in excess, ale, of all alcoholic drinks, tends pre-eminently to produce plethora, arising from an increase of fat, and to give rise to the peculiar transformation of the muscular system, known under the technical name of fatty degeneration. The muscles under this condition become converted in some measure into fatty substance, and lose their contractile force to a serious extent. Of all the muscles the heart often suffers most this change, and hence softening of the heart, impairment of the circulation, and serious symptoms arising from this failure, are common amongst great beer-drinkers. The blood also undergoes a change, losing its natural plastic properties, and becoming unusually fluid, so that wounds inflicted on the body of the ale-soaker heal with difficulty, and often when slight produce serious results. Ale-drinking, in short, when carried to an excess, as to the extent of three or four pints or more daily, is one of the most certain destructives of health and life.

See also *Beer, Hops, Malt, Porter.*

ALEC, a form of brine used by the ancient Romans. It was the dregs of a peculiar form of gamen or brine, made by mixing the intestines of small fishes with a large quantity of salt. After long exposure to the sun, and frequent stirring, a thick pulp was the result, which was strained through a wicker-work or basket: the thick residue was called alec, and was used as an inferior brine.

ALEWIFE, the common name given to a fish used as a food in America. It is in fact a herring, belonging to the genus *Clupea,* species *serrata.* It is sometimes called Aloof.

ALICA, a food used by the ancients. It is difficult to say whether it was a grain or a preparation from a grain. Many writers speak of it as a sort of wheat; but it seems rather to have been a kind of meal made into flummery and eaten with milk. Salmasius says, that Alica was one of the *chondroi* of the Greeks. The chondros was grain broken into large fragments, freed only from the husks, not ground.

(*To be continued.*)

